# Evaluation of Changes in Optical Coherence Tomography (OCT) Biomarkers After Oral Eplerenone Therapy in Chronic Central Serous Chorioretinopathy and Their Correlation to Functional Visual Outcome

**DOI:** 10.7759/cureus.98350

**Published:** 2025-12-02

**Authors:** Priyadarshini Mishra, Bruttendu Moharana, Shanmugasundaram Palanisamy, Bhagabat Nayak, Bijnya B Panda, Sandip K Sahu, Sucheta Parija

**Affiliations:** 1 Ophthalmology, All India Institute of Medical Sciences, Bhubaneswar, Bhubaneswar, IND

**Keywords:** chronic central serous chorioretinopathy, ellipsoid zone integrity, eplerenone, subretinal detachment, subretinal fluid

## Abstract

Purpose: This study aimed to assess the efficacy of the mineralocorticoid receptor antagonist eplerenone on chronic central serous chorioretinopathy.

Methods: In this prospective observational study, patients diagnosed with chronic central serous chorioretinopathy fulfilling the inclusion criteria and exclusion criteria were recruited. The best-corrected visual acuity (BCVA) on baseline was noticed, and optical coherence tomography (OCT) parameters (central macular thickness (CMT), subfoveal choroidal thickness (SFCT), subretinal fluid (SRF) height and base, ellipsoid zone (EZ) integrity) were measured at baseline. Participants were then advised about starting the eplerenone 25 mg tablet, and after one month, if serum K+ is <5 mmol/L, the dose was increased to 50 mg for two months. The same variables were assessed after three months.

Results: In this study, BCVA, SFCT, and SRF base changes from baseline to three months were statistically insignificant (p=0.061; p=0.354; p=0.577). However, CMT (p=0.016) and SRF height (p<0.001) have reduced significantly from baseline to three months. EZ integrity (p=0.009) showed significant changes from baseline to three months. On correlating BCVA changes with OCT parameters, findings were insignificant, but had a negative correlation for CMT (r=-0.030), SRF height (r=-0.083), and SRF base (r=-0.246).

Conclusion: Despite insignificant changes in BCVA, SFCT, SRF base, and EZ integrity, eplerenone showed a significant effect in reducing CMT and SRF height, which contributes to minimal improvement in visual acuity.

## Introduction

Central serous chorioretinopathy (CSC) is a disorder characterized by serous retinal detachment (SRD) and/or retinal pigment epithelial (RPE) detachment at the posterior pole [1-4]. The main risk factors are being male, being in the third to fifth decades of life, experiencing stress, smoking, exhibiting type A behavior, and other stress-related factors [2,5]. It has been reported that the overall incidence rate is higher in men than in women, with a reported ratio of 3:1 to 9:1 [1,5]. Usually, CSC is a self-limiting condition that spontaneously resolves in three months, so often observation is the first line of therapy for acute presentation. Later on, if not resolved spontaneously, it may turn into chronic CSC [1,2]. Even though the exact pathogenesis is not clear, CSC patients showed increased choroidal thickness in optical coherence tomography (OCT). A choroidal vascular abnormality, including vascular dilatation and hyperpermeability, was demonstrated through indocyanine green angiography in CSC patients [1]. It is also reported that the presence of mineralocorticoid receptor (MR) in the retinal ganglion cell layer, Müller cells, and inner nuclear layer is responsible for retinal fluid homeostasis regulation through aldosterone by upregulating ion and water channels [1,6]. Some studies reported that spironolactone is effective for faster fluid absorption in CSC than the natural course [1,6]. There are also studies reporting the effectiveness of the MR antagonist eplerenone in facilitating faster recovery from CSC [1,3]. Melatonin is an endogenous hormonal substance that is a neuromodulator and is also biosynthesized in the retina. Melatonin intake showed significant improvement in CSC patients [4].

CSC is one of the most common retinal conditions for visual impairment in one-third of cases, progressing to the chronic form. Eplerenone is a highly selective MR antagonist with lower affinity to other steroid receptors, thus minimizing sex hormone-related adverse effects and making it suitable for long-term use. The existing literature shows different results in different studies. Also, very few Indian studies were found. So, there is a need for further studies. We will look at OCT biomarkers, which can be measured accurately and are reproducible, to prognosticate the functional visual outcome after this treatment.

## Materials and methods

This prospective observational cohort study was conducted in the Department of Ophthalmology of All India Institute of Medical Sciences, Bhubaneswar, India, between June 2021 and December 2022 after obtaining approval from the institute's Institutional Ethics Committee (approval number: T/IM-NF/Ophthal/21/47). The primary aim of the study was to evaluate the changes in OCT biomarkers in patients with chronic CSC following oral eplerenone therapy and to correlate these changes with functional visual outcomes.

Study population

All consecutive patients diagnosed with chronic CSC, defined as persisting for more than three months in duration, were screened for eligibility. Patients aged 18 years or older, who were able to provide written informed consent, and who demonstrated subretinal fluid (SRF) beneath the fovea on OCT with leakage confirmed on fundus fluorescein angiography (FFA) were included. Additional inclusion criteria were OCT signal strength greater than 7, clear ocular media permitting good fundus visualization and OCT image acquisition, and a best-corrected visual acuity (BCVA) ranging between 0.3 and 1.0 logarithm of the minimum angle of resolution (LogMAR).

Exclusion criteria

Patients with hazy media due to corneal opacity, cataract, or vitreous hemorrhage, as well as those with a prior history of any treatment for CSC, were excluded. Individuals with concomitant ocular diseases affecting the macula, including age-related macular degeneration, diabetic macular edema, macular edema secondary to retinal vein occlusion, or polypoidal choroidal vasculopathy, were also excluded. Systemic exclusions comprised pregnancy or lactation, elevated serum potassium levels (≥5.0 meq/L), impaired renal function (serum creatinine >2.0 mg/dL in men and >1.8 mg/dL in women or creatinine clearance <50 mL/min), type 2 diabetes with microalbuminuria, and concomitant use of potassium supplements, potassium-sparing diuretics, or potent CYP3A4 inhibitors. Patients with contraindications identified during comprehensive medical evaluation, such as severe renal or hepatic dysfunction or known allergy to eplerenone, were not considered for enrollment.

Sample size calculation

The sample size was determined using the formula for comparing two means and an estimated mean difference of 46; the calculation at a 95% confidence level and an 80% power yielded a required sample size of 26 subjects.

Imaging modality

OCT images were acquired using Cirrus HD-OCT (Zeiss, Oberkochen, Germany). A 512×128 macular cube scan, five lines of raster scans, and enhanced depth imaging (EDI) scans were obtained, and central macular thickness (CMT) was measured automatically via the OCT software. SRF height under the fovea was measured manually on OCT scans using the linear measurement tool by drawing a perpendicular line between the neurosensorial retina and the inner edge of the RPE, and the maximum measurement (in microns) was reported. SRF basal diameter was manually measured from the widest point of the anechoically visible SRF on the spectral-domain OCT (SD-OCT). Choroidal thickness was assessed with the EDI scans and was measured from the posterior surface of the RPE to the choroidoscleral junction in the center of the fovea.

Statistical analysis

All the data were entered in a Microsoft Excel spreadsheet (Microsoft Corp., Redmond, WA, USA) and analyzed using IBM SPSS Statistics for Windows, V. 20.0 (IBM Corp., Armonk, NY, USA). The Shapiro-Wilk W test was used to assess the normal distribution of the variables, and p-values of <0.05 indicated that the tested data follow a non-normal distribution. Because of the non-normal distribution, a non-parametric test for related samples was used. The categorical variables were analyzed using Friedman's two-way analysis of variance by rank. The nominal data were analyzed using Cochran's Q test. The Spearman rho correlation analysis test was used to correlate BCVA with OCT parameters. A p-value of <0.05 was considered significant. 

## Results

This study has 26 participants with clinically diagnosed chronic CSC (26 eyes), with a mean age of 44.54 years, a male predominance of 80.8%, and a male-female ratio of 4:1. Of the participants, 65.4% had involvement in the right eye, while 34.6% had involvement in the left eye. Regarding habits, 38.4% of the participants were smokers, 50% of the participants had an alcohol consumption habit, and 19.2% were tobacco chewers. Regarding sleep, 20% of the participants slept less than six hours, 60% slept between six and eight hours, and 20% slept more than eight hours. Among the participants, 23% had diabetes mellitus type 2, and 34.6% had hypertension. This study presents a comprehensive analysis of clinical and OCT parameters in patients evaluated over a period of three months (Table 1).

**Table 1:** Demographic data and habits of the participants

S. no.	Variables		N=26	Frequency (%)
1	Gender	Male	21	80.8
		Female	5	19.2
2	Eye involved	Right	17	65.4
		Left	9	34.6
3	Habits	Smoking	10	38.4
		Alcohol	13	50
		Tobacco	5	19.2
4	Sleep hours	<6 hours	4	20
		6-8 hours	18	60
		>8 hours	4	20
5	Systemic diseases	Diabetes mellitus (type 2)	6	23
		Hypertension	9	34.6
		Cardiovascular disease	Nil	-
		Chronic kidney disease	Nil	-

One of the key metrics analyzed was BCVA, measured using LogMAR values. At baseline, the mean BCVA was 0.68, which improved to 0.48 at three months. The change from baseline to three months showed significant improvement (p=0.061), with the overall progression being highly significant (p<0.001). This indicates a steady and meaningful improvement in visual function over time. The CMT of participants had a baseline value of 334.69 µm to 237.81 µm at three months. The reduction became statistically significant by the third month (p=0.016) and showed a downward trendline, suggesting that the structural restoration of the retina accompanied the improvement in visual acuity. The subfoveal choroidal thickness (SFCT) of the participant's values remained relatively stable from 255.31 µm at baseline to 251.46 µm at three months (p=0.354). SFCT did not show a significant change over time, which suggests that SFCT may be less responsive or less closely associated with the functional improvements seen in vision. The SRF was measured for both the height and base length and showed a considerable reduction over time. The SRF height decreased significantly from 189.65 µm to 52.62 µm (p<0.001), and the base length dropped from 3759.27 µm to 717.88 µm. These reductions reflect the resolution of fluid accumulation, a key indicator of disease regression (Table 2).

**Table 2:** Analysis of vision and different OCT parameters at baseline and three months Friedman's two-way analysis of variance by ranks; a p-value of <0.05 is considered significant. CMT: central macular thickness; SFCT: subfoveal choroidal thickness; SRF: subretinal fluid; BCVA: best-corrected visual acuity in LogMAR values; OCT: optical coherence tomography

Variable (N = 26)		Mean±SD	Friedman's two-way analysis of variance by ranks		
			Test statistics	P-value	
BCVA	Baseline	0.68±0.16	0.865	0.061	<0.001
	Three months	0.48±0.24			
CMT (µm)	Baseline	334.69±151.54	2.327	0.016	
	Three months	237.81±89.26			
SFCT (µm)	Baseline	255.31±37.92	-0.692	0.354	
	Three months	251.46±40.32			
SRF height (µm)	Baseline	189.65±128.01	4.519	<0.001	
	Three months	52.62±84.76			
SRF base (µm)	Baseline	3759.27±5801.26	8.058	<0.001	
	Three months	717.88±1149.70			

Ellipsoid zone (EZ) integrity, an important marker of photoreceptor health, also showed progressive improvement over the course of the study. At baseline, only 34.6% of the patients had an intact EZ. This proportion increased to 57.7% at three months. The overall improvement in EZ integrity was statistically significant (p=0.009) by the end of the third month, highlighting a parallel structural recovery in the outer retinal layers (Table 3).

**Table 3:** Analysis of EZ integrity at baseline, one month, and three months Cochran's Q test; a p-value of <0.05 is considered significant. EZ: ellipsoid zone

EZ integrity (N=26)	Intact	Disrupted	Cochran's Q test statistics	P-value
Baseline	9	17	0.231	0.009
3 months	15	11		

Despite the noticeable improvements in both visual and structural parameters, the correlation analyses revealed only weak relationships between BCVA and the OCT measurements. At three months, correlations remained weak and statistically not significant across all the OCT parameters. This suggests that while OCT features do improve with time, they may not directly predict visual acuity outcomes, implying that other factors, such as photoreceptor health or neural processing, also play crucial roles in vision restoration (Table 4).

**Table 4:** Correlation analysis between BCVA and OCT parameters at baseline and three months Spearman's Rho correlation analysis; a p-value of <0.05 is considered significant. CMT: central macular thickness; SFCT: subfoveal choroidal thickness; SRF: subretinal fluid; BCVA: best-corrected visual acuity in LogMAR values

Changes in clinical variables at baseline and 3 months		CMT (µm)	SFCT (µm)	Height of SRF (µm)	Length of SRF base (µm)
BCVA in total eyes (N=26)	Correlation coefficient (r)	-0.030	0.245	-0.083	-0.246
	P-value	0.885	0.227	0.688	0.225

## Discussion

In this study, 26 participants were recruited. Among these participants, nearly 80% were males with a mean age of 44 years, 38% were smokers, and 20% had disturbed sleep patterns and stress as their risk factor, similar to that of previous studies by Takeshima et al. [2], Chrapek et al. [5], Bousquet et al. [6], Bousquet et al. [3], Gramajo et al. [4], Gergely et al. [7], and Fraenkel et al. [8].

In this study, the CMT mean value was 334.69 µm at baseline, which reduced to 237.81 µm by the end of three months after an oral therapy of eplerenone 25 mg tablet for one month. The difference in CMT reduction is 96.88 µm. Most participants (90%) showed complete resolution in three months. In a previous study, Bousquet et al. [3] evaluated predictive factors in non-resolving CSC cases treated with mineralocorticoid receptor antagonists (eplerenone vs. spironolactone). They reported a reduction in CMT from 383.2 µm to 294.7 µm (mean reduction: 88.5 µm), which is comparable to the reduction observed in our study (96.88 µm). Meanwhile, the effect of eplerenone in SFCT is very little and not significant either (p=0.354), which also correlates with previous studies by Bousquet et al. [3], Gergely et al. [7], and Pichi et al. [9]. In terms of SRF, there is a drastic reduction in the means of both height and base, which contributes to visual progression. In our study, the reduction of the SRF base is 3041.59 µm (p=0.577), which is statistically insignificant, and the height is 137.03 µm (p<0.001). There are MR present in retinal Müller cells. Aldosterone binds to MR and increases the potassium channel expression and inflow of potassium into the Müller cells, a process referred to as "siphoning of potassium". This, in turn, increases the aquaporin-4 (AQP4) water channel, which pumps the fluid out of the Müller cells and reduces edema, and maintains retinal homeostasis [9]. In CSC, these AQP4 channels were shifted to the base, and fluid enters the subretinal space [9,10]. Eplerenone, a derivative of spironolactone, has enhanced selective binding to MR and reduces the fluid shift. In terms of SRF, both spironolactone and eplerenone are equally effective [9]. Pichi et al. [9] and Felipe et al. [11] reported in a study that both mineralocorticoid antagonists were compared with placebo and there was also no statistically significant improvement in visual acuity in all three groups. Pichi et al. [9] have also reported that eplerenone showed nearly 91% of SRF reduction from baseline. In our study, there is also a nearly 90% reduction in SRF. These findings were correlated with previous studies by Schwartz et al. [12]. Pichi et al. [9] also reported that some reduction in size, nearly 9 microns, was noticed in Sattler's layer of choroidal large vessels, but insignificant SFCT reduction, similar to that of our study. In the VICI trial by Lotery et al. [13], the authors have reported that eplerenone has no role in the CSC condition. In contrast, eplerenone showed better results in our study and also in studies by Gergely et al. [7] and Pichi et al. [9] and in a survey among retina specialists by Venkatesh et al. [14]. Eplerenone was preferred for the treatment. There are no adverse events documented with eplerenone use. In our study, no adverse events were noticed. The EZ is disturbed in 65.4% of the participants, and after three months, it's reduced to 42.3%, as a result of the resolution of the SRF, but photoreceptor health is disturbed in these participants, resulting in poor vision improvement [15]. 

## Conclusions

This study highlights the promising impact of eplerenone on chronic CSC. The significant reduction in both CMT and SRF demonstrates the efficacy of the treatment, aligning with previous studies. The visual acuity improvements, though not strongly correlated with OCT parameter changes, suggest that other factors, like photoreceptor health, play crucial roles in vision restoration. Despite the varied findings in the literature, this study reinforces eplerenone's utility in CSC management without adverse events, offering a reliable option for clinical practice.
